# Common variable immunodeficiency (CVID) with granulomatous interstitial lung disease (GLILD) and SARS COVID-19 infection: case report and review of literature

**DOI:** 10.1186/s13223-021-00600-y

**Published:** 2021-09-26

**Authors:** Debendra Pattanaik, Shaunah Ritter, Joseph Fahhoum

**Affiliations:** 1 Division of Rheumatology and Immunology, Department of Medicine, University of Tennessee Health Sciences Center, Room G326, 956 Court Ave, TN 38163 Memphis, United States; 2 Division of Allergy and Clinical Immunology, Le Bonheur Children’s Hospital, University of Tennessee Health Sciences Center, TN 38163 Memphis, United States

**Keywords:** CVID, GLILD, COVID-19

## Abstract

**Background:**

We present a case of CVID complicated by granulomatous interstitial lung disease (GLILD). This patient clinical course was further complicated by COVID-19 infection. This is only the 2nd known case report of COVID 19 in CVID with GLILD. The clinical course and outcome of COVID 19 infection with common variable immunodeficiency (CVID) and GLILD is not well known.

**Case presentation:**

Our patient met the clinical features of CVID secondary to low IgG/IgA, recurrent infections, and failure to respond to pneumococcal vaccination. He was treated with monthly maintenance IVIG therapy. Our patient also was diagnosed with co-existing GLILD that despite IVIG treatment was progressing. The patient needed to be started on Rituxan and Mycophenolate mofetil to achieve control but unfortunately became infected with COVID19 delaying his treatment for GLILD. Our patient only suffered from mild COVID 19 infection and was able to make antibodies to this. We believe severe infection was avoided as his CVID was well controlled with IVIG therapy despite progression of his granulomatous interstitial lung disease.

**Conclusion:**

In conclusion, our patient with CVID with co-existing biopsy proven granulomatous interstitial lung disease despite being very high risk for severe COVID 19 infections only had mild infection. This was believed to be due to well controlled CVID with IVIG therapy.

## Background

Immunodeficiency and interstitial lung disease are mentioned as risk factors for unfavorable outcome in COVID-19 infection [1, 2]. However, there is limited data on the outcome of COVID-19 infection in this subset of patients especially with CVID. There are cases of fatal outcome in CVID patients who had coexisting underlying lung disease and other comorbidities [3, 4]. GLILD is an uncommon complication in CVID patients leading to progressive lung disease [5]. Our case shows an example of a patient with CVID complicated by granulomatous interstitial lung disease who became infected with COVID19.

## Case

**Fig. 1:**
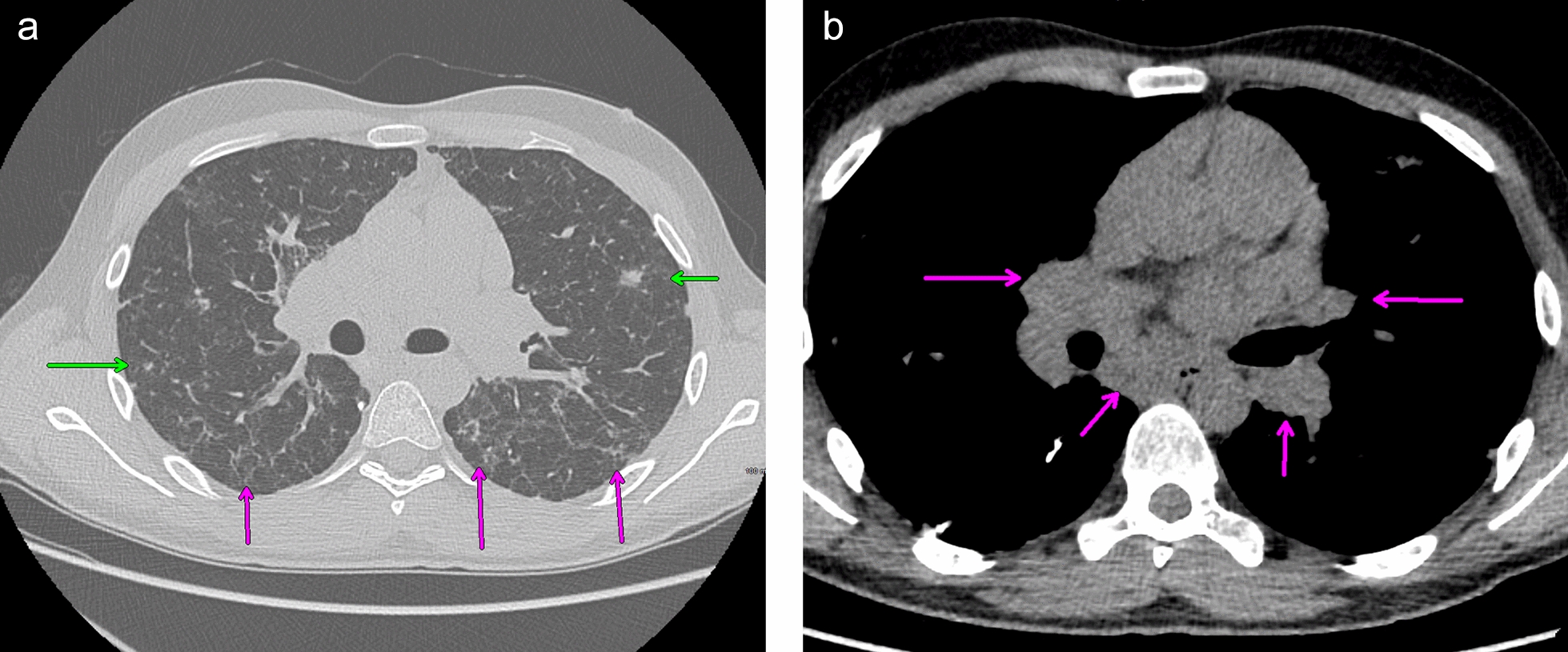
**a** CT chest showing scattered nodular (green arrow) and reticular densities (magenta arrow). **b** CT chest showing bilateral hilar and subcarinal lymphadenopathy (arrows)

A 27-year-old Caucasian male patient was initially evaluated in the allergy and immunology clinic in January 2019 for immunodeficiency. His initial symptoms started in June of 2017 when the patient started having recurrent episodes of pneumonia that were treated by his primary care physician (PCP). Incidentally at this time he was found to also have thrombocytopenia. He complained primarily of recurrent productive cough with green discharge over the past 1½ years. His other symptoms included: intermittent headaches, sinus pressure, nasal congestion, rhinorrhea, intermittent diarrhea, fatigue, and loss of smell but denied fever, chills, and night sweats. The patient had initially lost around 10–11 pounds at the start of his disease course. His family history was pertinent for a brother who also had thrombocytopenia and died from a brain aneurysm. Secondary to the above the patient was referred to hematology for further work up. Hematology completed further investigation during the Summer of 2017. This included monitoring of his platelet counts over a 12 month which showed varying levels between 91 and 108 × 10^9^/l. The rest of his CBC with differential, complete metabolic panel and urinalysis were normal. Quantitative immunoglobulin panels were completed four times over an additional 12-month period revealing the following: IgG 256–308 mg/dl (700–1600), IgM 9–25 (40–230), IgA 23–33 (70–400). A CT chest and abdomen revealed mediastinal and upper abdominal lymphadenopathy, splenomegaly, and multiple pulmonary nodules. Secondary, to his hypogammaglobulinemia he was vaccinated with pneumovax by the hematologist which included 2 doses, 1 month apart in May 2018 and June 2018. His findings on imaging made hematology concerned about tuberculosis, fungal infections, malignancy, and granulomatous disease such as sarcoidosis as a potential underlying cause. He was referred to pulmonary clinic in July 2018 for a lung biopsy. During his work up pulmonary function tests showed moderate reduction of airflow (FEV1/FVC: 59%), normal vital capacity and moderate reduction of diffusion capacity. These findings were certainly consistent with obstructive lung disease. He underwent bronchoscopy and ultrasound guided endobronchial lymph node biopsy twice in the summer of 2018. A Bronchoalveolar lavage (BAL) with multiple node biopsies were negative for bacterial, fungal and TB as a cause of lung disease. Testing was also negative for malignancy. He was started on fluticasone furoate, umeclidinium and vilanterol for further management with improvement in FEV1/FVC ratio (85%). However, part of his work up included an aspergillus galactomannan test which ended up being positive leading to pulmonary to start him on oral isavuconazonium sulfate to treat him for aspergillus infection. His antifungal treatment was continued for 5 weeks but was stopped in November 2018 secondary to side effects (elevated liver enzymes, nausea, and vomiting). Upon his follow up a repeat CT chest was ordered in December 2018 showing progression of multifocal nodularity in his lung and unchanged mediastinal lymphadenopathy. (Fig. 1a, b). Secondary to this he underwent VATS guided lung biopsy of his superior right lower lung segment in January 2019.

**Fig. 2:**
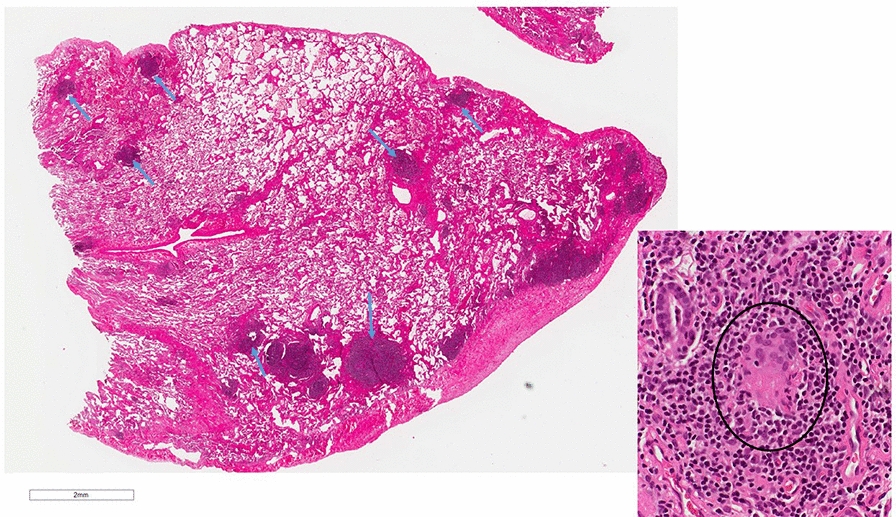
Lung biopsy showing lymphoid hyperplasia (arrows) and giant cell (inset)

When the patient presented to A/I clinic a physical examination showed the following: vital signs: 110/80, P: 64/minute, temp: 97, RR: 18/minute. ENT exam showed bilateral swollen pale nasal turbinate with light yellow drainage. The rest of his physical examination was unremarkable. Repeat laboratory testing was ordered in January 2019 and showed a platelet count of 96 K. The following tests were all negative CMP, urinalysis, stool ova and parasites, and HIV test. An Immunoglobulin panel showed an IgM 10 (40–230 mg/dl), IgG 520 (700–1600 mg/dl), IgA 26 (70–400 mg/dl), IgE  <  2 (0–158 mg/dl). Lymphocyte subsets are as followed: absolute lymphocyte: 1126 (1000–4000), CD3: 935 (960–2600) [83% (61–84)], CD4: 586 (540–1660) [52% (32–60)], CD8: 304 (270–930) [27 (13–40%)], CD19: 79 (122–632) [7% (3–22)], CD16  +  56: 90 (70–480) [8% (3–22)]. As the next step of work up a lymphocyte mitogen screen showed a low to PHA and a normal Con A and Pokeweed Mitogen. A sinus X ray was remarkable for left maxillary sinus disease showing mucosal thickening. Despite being vaccinated the patient had poor pneumococcal antibody titers with only 2/14  ≥  1.3 μg/ml being responsive. His tetanus antibody titer was in the protective range of 1.3. The patient eventually did have a lung biopsy that showed lymphoid hyperplasia with interstitial fibrosis, patchy foci of organizing pneumonia, rare giant cells/histiocytes, acute bronchopneumonia and occasional fibrin exudates. Overall, the interstitial findings are compatible with the CVID-related interstitial lung disease, granulomatous lymphocytic interstitial lung disease (GLILD). Given the extensive lymphoid component, MALT lymphoma was considered; however, the immunohistochemistry profile, flow cytometry and molecular studies (per report) show no evidence of a clonal B-cell process. (Fig. 2). Based on these findings, allergy/immunology service confirmed the diagnosis of CVID with coexisting immune thrombocytopenia and GLILD. The patient had been started on treatment with IVIG 30 g every 4 weeks based on his initial immunoglobulins by hematology and this was continued by us. We obtained repeat quantitative IgG after therapy and the level was 600 (600–1640 mg/dl) on 10/1/2019. We subsequently increase his IVIG dose to 50 g every 4 weeks because of decreasing lung function and decreased platelet count. The patient currently remains infection free since November 2018. His follow up chest X ray and CT chest in January 2020 showed further widespread lymphadenopathy and nodular lung densities. Allergy service referred him to rheumatology to begin treatment with Rituximab and mycophenolate mofetil for GLILD. While waiting for treatment with Rituxan, he developed high grade fever, headache, and body ache. He subsequently tested positive for COVID 19 (RT-PCR) through nasal swab. He slowly recovered without any complication at home. Of note the patient did have positive SAR-Cov-2 total antibody test: 207.3 [(0–0.9 index) (Roche ECLIA)] about 10 weeks after he tested positive for COVID-19. The patient eventually was able to start Rituximab infusion of 375 mg/m^2^ weekly for 4 weeks and completed four cycles of therapy without complication. He is now just on maintenance mycophenolate mofetil 1 g and monthly IVIG and is doing well.

## Discussion

Our patient fulfilled the diagnostic criteria for CVID [6]. He suffered recurrent pulmonary and sinus infections accompanied by marked decrease in IgG and IgA level as well as poor vaccination response to pneumovax 23. GLILD is a distinct clinical entity with unique radiologic and histological features seen in 20% of patients with CVID. GLILD is associated with a lymphocytic infiltrate and/or granuloma in the lung, and in whom other conditions have been considered and excluded [5]. 21% of CVID patients complicated by GLILD had damaging mutation in genes e.g., TNFRSF13B, CTLA4, KMT2D, BIRC4 known to cause CVID [8]. The diagnosis is usually made by CT chest followed by an open lung biopsy [5] However, CT findings are not specific and include pulmonary nodules, ground-glass opacities, hilar and/or mediastinal lymphadenopathy, and reticulation that be seen in other etiologies [5]. That is why an open lung biopsy is essential to exclude other infectious and malignant conditions. Histologic findings with GLILD include granulomatous inflammation, peribronchiolar lymphoid proliferation, interstitial lymphoid proliferation, with a CD4  +  T-Cell predominance. We were able to confirm that he had GLILD based on his prior diagnosis of CVID and CT chest and histologic findings. There was a delay in diagnosis of CVID and GLILD for about year and half until he was seen by allergy and immunology. Such delay in diagnosis is not uncommon for CVID cases [7]. Currently there is no consensus on the treatment of GLILD aside from continuing IVIG therapy, though corticosteroids have been used in the past [5]. Recent literature does support combination chemotherapy using Rituximab with azathioprine or mycophenolate mofetil as the first line therapy [8]. A summary of treatment regimens is summarized in Table 1. Based on the literature above which is the largest cohort of CVID patient with GLILD, we decided to treat using rituximab and mycophenolate mofetil without corticosteroid. There is risk of opportunistic infection with immunosuppressive treatment. Pneumonia and opportunistic infection e.g., pneumocystis, nontuberculous mycobacteria, varicella zoster and possibly progressive multifocal leukoencephalopathy have been reported [5, 8].

**Table 1:** Treatment of CVID-GLILD with immunosuppressive therapy

Agent	RTX [17]	RTX + AZA [8]	RTX + MMF [8, 18]
Number of patients	3	29	15
Regimen used	375 mg/m^2^ weekly × 4, then every 6 months	RTX: 375 mg/m^2^ weekly × 4, then every 4–6 months + AZA: 1–2 mg/kg/day	RTX: 375 mg/m^2^ weekly × 4, then every 4–6 months + MMF: 250–1000 mg twice daily (N = 14)
			RTX: 1 g × 2 dose 1 month apart + MMF: 1–2 g/day (N = 1)
Duration of follow up	24 months	16 months	16 months
Outcome	Improvement in CT chest and PFT	Improvement in CT chest and PFT, death = 1, relapse = 6	Improvement in CT chest and PFT, death = 1, relapse = 3
Adverse events	None reported	Reversible hepatotoxicity, intolerance, pneumonia	Pneumonia

*AZA* azathioprine, *MMF* mycophenolate mofetil, *RTX* rituxan

COVID19 is known to be high risk to patients with comorbid conditions such as chronic lung disease, age  >  65, and underlying immunodeficiency state etc. [1]. Patients with interstitial lung disease like ours are at an increased risk of hospitalization and ICU care [2]. Our patient was possibly at an even higher risk given the history of CVID and coexisting interstitial lung disease.

There is limited data on the outcome of COVID-19 infection in CVID patients based on case reports and small case series. Ho et al. looked at 16 patients with primary immunodeficiency (PID) who had COVID-19 infection and 9 of them were CVID patients [3]. Patients with PID can have a range of disease severity from mild to severe infection. However, a portion in this study showed that 25% (4/16) died making the mortality rate in PID higher compared to the general population. In the same study, 2/16 patients with CVID and COVID19 infection died but each had associated lung disease such as bronchiectasis and interstitial lung disease that was unspecified [3]. The patients who were on maintenance IVIG replacement therapy and those without preexisting autoimmune/inflammatory disease had better outcomes. Two other published case reports showed successful recovery from COVID-19 infection in patients with PID. Both patients had bronchiectasis and happened to receive additional IVIG during hospitalization. The authors attributed their recovery to having stable IgG level with their maintenance IVIG treatment prior to infection [9, 10] However another subject with CVID had a fatal outcome from COVID-19 infection from secondary bacterial infection despite multiple courses of IVIG infusion while being hospitalized. This patient was off their maintenance IVIG therapy with low IgG levels upon infection and hospitalization [4]. The comparison of these cases shows importance of having good control of CVID prior to infection with COVID19. Our patient did well as he had uninterrupted IVIG infusion throughout the whole time and normal IgG levels. In a small study IVIG administration was given to patients without CIVD who had severe COVID-19 infection and had not responded to initial treatment and showed decrease mortality [11]. Some of the current IVIG products in United States (Gammunex-C 10% and Flebogamma 5% DIF) have cross reacting antibodies against SARS-CoV-2 from other coronavirus families e.g., SARS-CoV and MERS-CoV [12]. The authors suggest presence of cross-reacting SARS-CoV-2 antibodies along with immunomodulatory and anti-inflammatory effect of IVIG may help severe COVID-19 infection [12].

Like our patient, some CVID patients mount SARS-CoV-2 detectable antibody responses though the duration and significance of it is not clear at this point per that author [3]. It would be difficult to tell without prior testing if our patient had cross reacting antibodies.

Patients with CVID may be at a higher a risk for severe disease compared to patients with X linked agammaglobulinemia (XLA). In a small case series involving 7 patients (5 with CVID and 2 with agammaglobulinemia) Quinti et al. [13] noted that CVID patients had a more severe disease course compared to the XLA group despite having similar baseline and maintenance Ig levels. This is suspected to be due to lack of B lymphocytes in patients with XLA vs having dysfunctional B lymphocytes in CVID patients. CVID patients were also noted to have higher levels of inflammatory markers e.g., C-reactive protein, fibrinogen, D-dimer, IL-6, IL-8, and TNF-α compared to XLA patients [3]. The Bruton Tyrosine Kinase (BTK) protein mediates signaling of the viral ssRNA-sensing toll-like receptor pathway [14]. Increase in monocyte BTK activation was found during severe COVID-19 infection and BTK inhibitors could potentially be used to treat severe COVID-19 related inflammation and lung injury [15, 16]. It is likely B lymphocytes play a role in COVID-19 induced inflammation [13].

## Conclusion

In conclusion our CVID patient whose course is complicated with GLILD recovered from COVID-19 infection at home uneventfully compared to other cases with comorbid pulmonary conditions. A recent abstract published showed a patient with CVID and GLILD on infliximab had  a mild disease course like our patient [16]. This further supports that it is likely that regular maintenance IVIG infusion may have prevented a bad outcome as well as immunomodulating medications helping prevention of the cytokine storm [16]. Of note our patient was also able to generate significant SARS-COVID-19 antibody response following infection. However, a general conclusion of the excellent outcome of COVID-19 infection in CVID with GLILD patients cannot be made just based on 2 case reports and need further study.

## Data Availability

Not applicable.

## Abbreviations

SARS: Severe acute respiratory syndrome
COVID-19: COronaVIrusDisease-2019
CVID: Common variable immunodeficiency
GLILD: Granulomatous interstitial lung disease
PID: Primary immunodeficiency
XLA: X linked agammaglobulinemia
IVIG: Intravenous immunoglobulins
BTK: Bruton tyrosine kinase
ICU: Intensive care unit
MALT: Mucosa associated lymphoid tissue
TB: Tuberculosis
BAL: Bronchoalveolar lavage
FEV: Forced expiratory volume
FVC: Forced vital capacity
VATS: Video assisted thoracoscopic surgery
RT-PCR: Reverse transcription polymerase chain reaction
RTX: Rituxan
AZA: Azathioprine
MMF: Mycophenolate mofetil
